# Orchestration of synaptic functions by WAVE regulatory complex-mediated actin reorganization

**DOI:** 10.1038/s12276-023-01004-1

**Published:** 2023-06-01

**Authors:** Kyung Ah Han, Jaewon Ko

**Affiliations:** 1 Department of Brain Sciences, Daegu Gyeongbuk Institute of Science and Technology (DGIST), 333 Techno Jungangdae-Ro, Hyeonpoong-Eup, Dalseong-Gun, Daegu, 42988 Korea; 2 Center for Synapse Diversity and Specificity, DGIST, Daegu, 42988 Korea

**Keywords:** Neuroscience, Synaptic transmission

## Abstract

The WAVE regulatory complex (WRC), composed of five components—Cyfip1/Sra1, WAVE/Scar, Abi, Nap1/Nckap1, and Brk1/HSPC300—is essential for proper actin cytoskeletal dynamics and remodeling in eukaryotic cells, likely by matching various patterned signals to Arp2/3-mediated actin nucleation. Accumulating evidence from recent studies has revealed diverse functions of the WRC in neurons, demonstrating its crucial role in dictating the assembly of molecular complexes for the patterning of various *trans*-synaptic signals. In this review, we discuss recent exciting findings on the physiological role of the WRC in regulating synaptic properties and highlight the involvement of WRC dysfunction in various brain disorders.

## Brain disorders: Multiple roles of protein complex need further exploration

Extensive research is needed to understand how the components of a critical protein complex at synapses influence the properties of neural circuits. Jaewon Ko and Kyung Ah Han, Daegu Gyeongbuk Institute of Science and Technology, South Korea, reviewed the roles of the five protein components in the WAVE Regulatory Complex (WRC), and how they organize synaptic properties and neural circuits. The WRC plays key roles in the dynamics and remodeling of actin, a major cytoskeletal protein critical for synapse formation and maintenance. The WRC also activates other protein complexes significantly involved in controlling actin activity. Each WRC component has distinct functions at synapses, such as regulating the growth of dendritic spines (protrusions on dendrites, the extensions of neurons that receive stimuli) and presynaptic assembly. Undoubtedly, dysfunction of the WRC components are associated with various brain disorders.

## Introduction

Actin, a major cytoskeletal element, is essential for a range of fundamental cellular processes in eukaryotes. Actin plays a key role in neuronal morphogenesis and synapse formation during nervous system development^1^. In mature neurons, actin is the most prominent cytoskeletal component in both presynaptic and postsynaptic compartments, including dendritic spines, which mediate most excitatory synaptic transmission in the brain^2–4^. In addition, actin cytoskeletal remodeling has been implicated in structural synaptic plasticity and is intimately linked to the proper operation of molecular machineries critical for synaptic functions involving a cohort of various actin-binding proteins that regulate the assembly and disassembly of actin filaments^5–7^. Notably, it has been established that the Arp2/3 (actin-related proteins-2 and -3) complex, an evolutionarily conserved actin nucleating hub, acts in conjunction with dozens of other nucleation-promoting factors to drive polymerization, organization, and recycling of the actin filament network^8^.

**Fig. 1:**
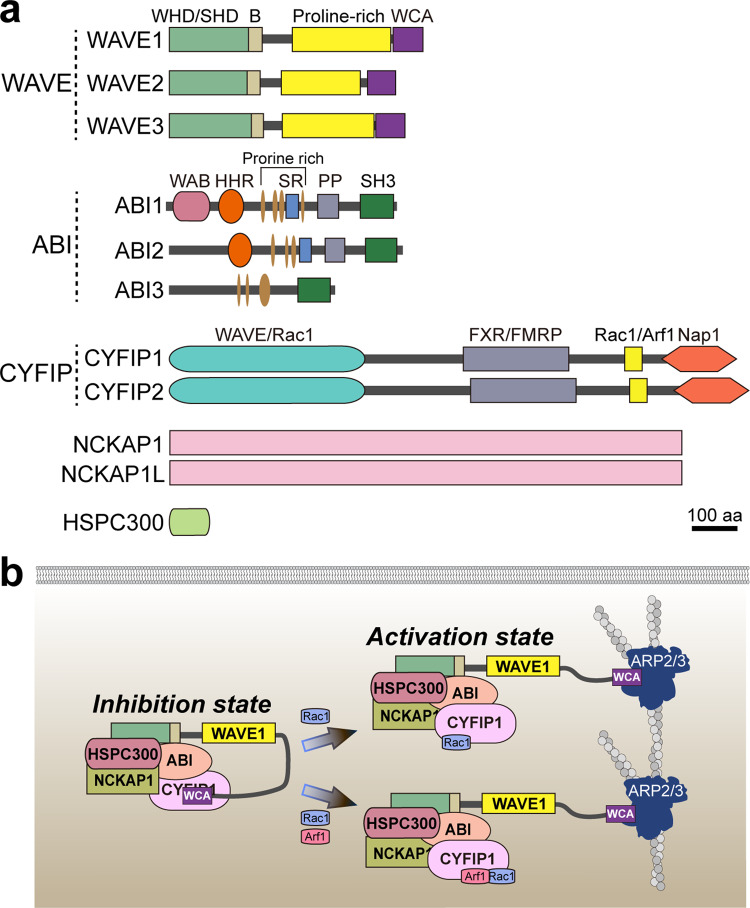
WRC subunit composition and assembly mechanism. **a** Schematic depicting WRC subunits and their homologs. The WRC is a five-subunit complex comprising the following protein families: ABI (ABI1, ABI2 or ABI3), WAVE (WAVE1, WAVE2, or WAVE3), Nap1 (Nckap1 or Nckap1L), CYFIP (CYFIP1 or CYFIP2), and HSCP300. aa amino acid, B basic domain, HHR Hox homology region, PP polyproline structure, SH3 Src homology 3 domain, SHD SCAR homology domain, SR serine/threonine-rich region, WAB WAVE-binding domain, WCA WASP homology 2-central-acidic, WHD WAVE homology domain. **b** Schematic illustration of two modes of WRC activation in actin polymerization. In the absence of Rac1 binding, WRCs exist in an autoinhibited state. Rac1 binding to the A site located at the N-terminus of CYFIP1 induces WRC activation. This destabilizes the meander sequence of WAVE1, which is critical for autoinhibition, inducing a conformal change that triggers the release of the WCA sequence, making it accessible to the ARP2/3 complex. In contrast, Rac1 binding to the D site, located in the C-terminal region of CYFIP1, does not directly activate WRC but does increase the affinity for ARF1 binding between the D site of CYFIP1 and the W helix of the WCA domain of WAVE, allowing the WCA region of WAVE1 to activate ARP2/3.

Among nucleation-promoting factors, members of the WASP (Wiskott-Aldrich syndrome protein), neuronal WASP, and WAVE (WASP family verprolin homologous protein; also known as SCAR [suppressor of cyclic AMP [cAMP] receptor] family have been highlighted as ubiquitous regulators of actin cytoskeletal remodeling^9–11^. These proteins exist in a heteropentameric macromolecular complex (~400 kDa) known as the WRC (WAVE regulatory complex)^11,12^. The WRC is assembled from five different proteins—CYFIP (cytoplasmic FMR1-interacting protein; also known as Sra1), NAP (NCK-associated protein; also known as Nckap1), ABI (Abelson-interacting protein), HSPC300 (hematopoietic stem progenitor cell 300; also known as Brk1), and WAVE—that are all essential for WRC functions^11^ (Fig. 1a). Remarkably, for each component protein, there are homologous proteins that likely exhibit tissue-specific and/or cell-type-specific expression, employ distinct regulatory mechanism(s) and/or differentially activate Arp2/3-mediated actin polymerization, as elaborated below. The same is true for orthologous subunits in different organisms. The WRC is intrinsically inactive but is activated upon interaction with numerous cytosolic proteins, small GTPases, and transmembrane receptors, causing its translocation to the plasma membrane, where it activates the Arp2/3 complex^11^. Notable regulators include the Rho-family GTPase Rac1 and Arf GTPase Arf1, which allosterically relieve autoinhibition of the WRC by releasing the WCA (WASP homology 2-central-acidic) domain^13,14^ (Fig. 1b). Moreover, PIP_3_ [phosphatidylinositol-(3,4,5)-triphosphate] further enhances Rac1-mediated WRC activation^15,16^. Although our understanding is still incomplete, extensive research on the basic biology and regulatory mechanisms of the WRC has significantly contributed to our view of how actin networks are organized in eukaryotic cells.

In the current review, we describe the known functions of WRC components at neuronal synapses. We then discuss the role of the WRC in orchestrating key aspects of synapse development. Finally, we highlight the association of WRC dysfunctions with certain brain disorders and consider their ramifications. Owing to space constraints, we limit our presentation of background information on the WRC to neuroscience-related topics; recent review articles are available for those interested in greater detail^11,17^.

## The role of WRC components during nervous system development

Although the significance of the WRC in regulating actin dynamics and remodeling has gradually come to be appreciated, the roles of individual WRC components in various processes during nervous system development have not yet been convincingly defined. Because each of the five components—Cyfip1/Sra1, WAVE/Scar, Abi, Nap1/Nckap1, and HSPC300/Brk1—also mediates complex formation with other distinct proteins, it is possible to speculate that distinct functions of each WRC component shape concerted Arp2/3-mediated F-actin polymerization. In this section, we describe key observations involving each WRC component and their relation to actin remodeling processes in various contexts. We also highlight a subset of issues that require further clarification.

### Cyfip

The two evolutionarily conserved proteins, Cyfip1/Sra1 and Cyfip2/Pir121, that constitute the Cyfip protein family were initially identified as interacting partners of FMRP (fragile X mental retardation protein), an RNA-binding protein involved in the GTP-dependent translational control of synaptic proteins^18^. It was subsequently shown that Cyfip1 and Cyfip2, as Rac1 effectors, constitute the WRC^19–21^ and form a complex with eIF4E (eukaryotic translation initiation Factor 4E) modulated by BC1 (brain cytoplasmic RNA 1)^22^, suggesting that Cyfip might provide a bridge between actin remodeling and translation. Moreover, BDNF (brain-derived neurotrophic factor) activates Rac1, causing Cyfip1 to dissociate from the FMRP-eIF4E complex (thereby antagonizing FMR1 [fragile X messenger ribonucleoprotein 1] functions) and associate with Rac1-WRC, leading to inhibition of translational repression^22^.

Both Cyfip1 and Cyfip2 are enriched at synapses of excitatory postsynaptic spines, where they regulate F-actin dynamics and dendritic spine development^23–25^. Overexpression of Cyfip1 or Cyfip2 enhances dendritic complexity and outgrowth, whereas haploinsufficiency of either result in abnormal dendritic spines^26,27^. Another study showed that both Cyfip1 and Cyfip2 are also localized at GABAergic synapses^28^. Strikingly, overexpression of Cyfip1 or Cyfip2 decreases inhibitory synapse structure and transmission, disrupting the excitatory-to-inhibitory (E/I) synaptic balance^28^. Similarly, postsynaptic loss of Cyfip1 increases inhibitory synapse size and strength, concomitant with the upregulation of GABA_A_ receptor β2/3 subunits and the synaptic adhesion protein Nlgn3 (neuroligin−3)^28^. Studies on Cyfip1 heterozygous (*Cyfip1*^+/-^) mice illustrate the postsynaptic role of Cyfip1, demonstrating altered synapse composition in the hippocampus of these mice, with decreased levels of SynGAP1 (a synaptic Ras GTPase activating protein 1) and GluA1 (an AMPAR [α-amino-3-hydroxy-5-methyl-4-isoxazolepropionic acid receptor] subunit) and increased levels of mGluR1/5 [metabotropic glutamate receptor 1 and 5], GluN2B (an NMDAR [N-methyl-D-aspartate receptor] subunit), and F-actin^29,30^. Cyfip1 also participates in the regulation of presynaptic nerve terminal size and vesicle release probability through its actions on the WRC downstream of Rac1^31^, whereas Cyfip2 regulates presynaptic short-term plasticity through its actions on presynaptic mitochondria in medial prefrontal cortical neurons^32^. However, it remains unclear how Cyfip proteins, which are present at both excitatory and inhibitory synapses, form complexes with Rac1, WAVE1, FMRP, and other proteins to bidirectionally regulate the synaptic E/I ratio. In addition, it is unknown whether the identified presynaptic roles of Cyfip proteins are universal across diverse brain regions.

Each Cyfip paralog also performs distinct functions. Analyses of Cyfip1/2-haploinsufficient mice showed that *Cyfip2*^+/−^, but not *Cyfip1*^+/−^, mice exhibit cell-type–specific defects in spine morphogenesis^27^. In line with this observation, Cyfip1 and Cyfip2 distinctively regulate retinal ganglion cell axon growth and guidance in zebrafish^33^. In particular, upon axon-axon contact, Cyfip2 is cotransported with RNPs (ribonucleoprotein particles) to the growth cone periphery, where it switches its association, dissociating from RNPs and associating with the WRC to regulate actin polymerization and filopodial dynamics^33^. Expression of Cyfip1 fails to rescue the axon-sorting defect phenotype associated with *Cyfip2* deletion^33^, indicating the nonredundant function of Cyfip paralogs during retinal development. This axon-segregating function of Cyfip2 likely relies on its binding to a subset of transmembrane receptors involved in processes that govern proper axon sorting^34^. It is also possible that Cyfip2 functions are distinct across diverse animal species (e.g., see ref. ^35^). Intriguingly, single-cell RNA sequencing analyses revealed that Cyfip1 is expressed in both neurons and astrocytes, whereas Cyfip2 is predominantly expressed in neurons^36^, a finding that warrants future studies using conditional KO (knockout) lines to systematically analyze the effects of neuron-specific or astrocyte-specific deletion. It is also important to consider differences in the post-transcriptional regulatory activities of Cyfip paralogs in analyses of phenotypes arising from cell-type-specific deletion^37^.

### Abi

Members of the Abi protein family, which includes Abi1, Abi2, and Abi3/NESH, were initially identified as substrates of c-Abl tyrosine kinase (with the exception of Abi3)^38–40^. Abi proteins form complexes with various proteins (e.g., Eps8, Sos1, or WAVE2) linked to the Rac1-orchestrated actin remodeling pathway that leads to enhanced Arp2/3-mediated actin nucleation^40–42^. Abi1 promotes tyrosine phosphorylation of several proteins, including Mena (mammalian enabled), BCAP (B-cell adaptor for PI3-kinase), Cdc3 and WAVE2, promoting localization of these proteins to specific subcellular compartments^43–46^. Intriguingly, Abi1 itself is phosphorylated at serine 88 by CaMKIIα (Ca^2+^/calmodulin-dependent kinase IIα), which is essential for Abi1-dependent modulation of spine morphogenesis^47^. Abi1, initially enriched in growth cones, is relocated to filopodia and dendritic spines and becomes restricted to the postsynaptic density matrix through interactions with ProSAP2/Shank3 (SH3 and multiple ankyrin repeat domains 3). Upon synaptic activation, it translocates to the nucleus through interactions with heterogeneous nuclear ribonucleoprotein K or is retransported to excitatory synaptic sites through interaction with the motor protein Kif26B^48–50^. In the nucleus, Abi1 forms a trimeric complex with Myc and Max transcription factors that facilitates the transcription of E-box-regulated genes, including epidermal growth factor receptors^48^. All three Abi proteins are characterized by their involvement in dynamic actin cytoskeleton remodeling, as demonstrated in various model organisms^42,48,51–56^, but whether and how each Abi paralog exerts distinct actions in the regulation of actin dynamics at synaptic sites remain unexplored.

### WAVE/SCAR

Three members of the WAVE protein family (WAVE1–3), also known as the SCAR family, possess modular domains that interact with the small GTPases Cdc42 or Rac1 or the adapter protein IRSp53, leading to activation of the Arp2/3 complex and subsequent actin remodeling and branching at the leading edges of cells^57^. WAVE1 is highly expressed in the brain, and its deletion abnormally alters the size of both presynaptic terminals and postsynaptic spines^58^. Moreover, *Wave1*-KO mice exhibit deficits in sensorimotor function, including impaired motor coordination and balance, reduced anxiety levels, and deficits in hippocampus-dependent learning and memory^59^. Because removal of WAVE1 impedes assembly of the WRC, it is likely that WAVE1 and its binding proteins, such as WRP (WAVE1-associated RacGAP protein) and profilin, form a localized hub that serves to properly regulate Rac signaling. Notably, as a kinase-anchoring protein, WAVE1 is linked to various protein kinases, including cAMP-dependent PKA (protein kinase A) and Cdk5 (cyclin-dependent kinase 5)^60,61^. Cdk5 phosphorylates three residues in WAVE1, an action that is reversed by stimulation of D1-type dopamine receptors and NMDARs^62,63^. An acute challenge of mice with cocaine following a 2-week course of cocaine administration and subsequent 2-week withdrawal period similarly dephosphorylates residues phosphorylated by Cdk5^60^. Moreover, WAVE1 is expressed in MSNs (medium spiny projection neurons) in the striatum; further analyses using D1-MSN-expressing, neuron-specific *Wave1*-KO mice showed that WAVE1 is essential for activity-dependent regulation of dendritic spine density and excitatory synaptic transmission selectively in D1-MSN neurons^60^.

Similar to WAVE1, WAVE2 is involved in maintaining dendritic spine density and size through a mechanism that involves an IRSp53-dependent pathway^64^; it also controls dysbindin-1-mediated dendritic morphogenesis^65^. Moreover, WAVE2 interacts with c-Abl tyrosine kinase, which is modulated by Abi1^66^. Notably, c-Abl-mediated tyrosine phosphorylation of WAVE2 is required for the actin remodeling activity of WAVE2^66^. WAVE3 is also phosphorylated by c-Abl, and this phosphorylated form of WAVE3 regulates lamellipodia formation and cell migration^67^. Puzzlingly, Abi1 is crucial for c-Abl-mediated tyrosine phosphorylation of WAVE2 but not WAVE3^66,67^, implying the presence of additional intermediate(s) that could be uniquely associated with WAVE3-containing WRCs. Because each WAVE paralog exhibits differential localization in growth cones^68^, it is plausible that complexes containing distinct WAVE orthologs have different roles during synapse development. Future analyses of the interactomes of each WAVE paralog in vivo will be required to better understand the conserved and divergent functions of the WRC.

### Nap1/NCKAP1

Nap1, also termed NCKAP1 (Nck-associated protein 1), was demonstrated to interact directly with Rac1 and the other WRC components, Cyfip1 and Abi1, to regulate WAVE1 activity, which is required for Arp2/3-mediated actin polymerization and branching at protrusive membrane edges and subsequent lamellipodial extension^54,69,70^. Nap1 is localized along lamellipodia and mediates cell migration and laminar-specific neuronal differentiation in the developing neocortex^55,71–73^. It is also responsible for remodeling the motility and adhesion machinery by forming complexes with OL-protocadherin^72^. Moreover, Nap1 expression in cortical neurons is upregulated by BDNF^73^, and its stability is modulated by interaction with HSP90 (heat shock protein 90)^74^. These studies clearly establish a role for Nap1 in developing neurons, but the function of Nap1 at mature stages of synapse development and details of the underlying mechanism remains unclear.

### BRK1/HSPC300

Brk1 (BRICK1)/HSPC300, the smallest component (~8 kDa) of the WRC, has been most extensively studied in the context of cytoskeletal remodeling in *Arabidopsis*^75,76^, with these studies suggesting a crucial role of HSPC300 in promoting Arp2/3 activity^77^. However, a study employing RNAi (RNA interference) showed that the effects of HSPC300 knockdown in cultured *Drosophila* S2 cells are modest relative to those observed with RNAi-mediated ablation of other WRC components^54^. Moreover, other studies have shown that HSPC300 is not required for the assembly of the WAVE complex in vitro or Arp2/3-mediated actin polymerization^42,78^. In the only currently available study, *Drosophila* HSPC300 was shown to regulate synaptic morphology at NMJs (neuromuscular junctions) by forming a complex with Rac1-WAVE proteins^79^. However, the lack of studies targeting vertebrate HSPC300 orthologs has hindered the establishment of the critical roles of these proteins in the WRC.

## Synaptic functions of the WRC

### Postsynaptic spine morphogenesis

Dendritic spines are morphologically diverse, protrusive structures studding from dendritic shafts that receive the most excitatory synaptic inputs^2,80,81^. They are almost exclusively enriched with F-actin, a polymerized form of actin filaments; notably, remodeling of F-actin governs excitatory synapse physiology. Numerous actin regulators that mediate the tight control of assembly and signaling in dendritic spines have been identified and shown to collectively orchestrate actin remodeling dynamics in conjunction with a host of spine-enriched activators and/or inhibitors that tune the activity of various small GTPases^82–84^. Arp2/3 is the most notable central regulator, and together with other F-actin regulators, such as formins and profilins, it drives actin filament assembly, dendritic spine maturation, and dendrite branching^81,85,86^. A subset of protein kinases provides phosphorylation-based regulation of the WRC^87^, altering its conformation in a manner that facilitates interactions with Arp2/3. The WRC is activated and relocated to membranes by the concerted actions of upstream factors, including Rac1 and other proteins, as well as by PIP_3_^11,88^. Rac1-mediated promotion of spine growth involves NMDA receptor activation and direct interaction with the WRC component Cyfip1^25^. In addition, PIP_3_ binds to the WRC component WAVE2 to regulate the formation of filopodia-like protrusion structures that project from spines during structural plasticity in hippocampal CA1 pyramidal neurons^89,90^. Furthermore, the WRC physically interacts with diverse synaptic receptors that likely transduce patterned extracellular signals into the intracellular compartment^34^ (Table 1; see also below for details). Whether the WRC-Arp2/3 axis operates similarly across diverse cell types and brain regions needs to be systematically investigated in future studies.

**Table 1:** WIRS-containing transmembrane proteins.

Gene name	Protein name	Sequences and positions of WIRS
PTPRS	Receptor-type tyrosine-protein phosphatase S	LATFCV (aa 1526–1531) LGSFDH (aa 1940–1945)
PTPRD	Receptor-type tyrosine-protein phosphatase delta	LGSFDH (aa 1904–1909)
PTPRF	Receptor-type tyrosine-protein phosphatase F	LGSFDH (aa 1899–1904)
EPHA3	Ephrin type-A receptor 3	LDSFLR (aa 707–712) ITTFRT (aa 910–915)
EPHA5	Ephrin type-A receptor 5	LDTFLK (aa 761–766)
EPHA6	Ephrin type-A receptor 6	LDSFLR (aa 759–764) IVSFLD (aa 926–931) FTTFDL (aa 987–992)
EPHA7	Ephrin type-A receptor 7	FTTFCS (aa 922–927)
EPHA8	Ephrin type-A receptor 8	LDTFLR (aa 721–726) FRTFSS (aa 812–817)
EPHA10	Ephrin type-A receptor 10	FSTFPS (aa 929–934) FPSFGS (aa 932–937)
EPHB1	Ephrin type-B receptor 1	LDSFLR (aa 705–710)
EPHB2	Ephrin type-B receptor 2	LDSFLR (aa 707–712) YTSFNT (aa 912–917) FTSFDV (aa 939–944)
EPHB3	Ephrin type-B receptor 3	LDSFLR (aa 719–724) YTTFTT (aa 924–929) FASFDL (aa 951–956)
EPHB4	Ephrin type-B receptor 4	LDSFLR (aa 701–706) FGSFEL (aa 933–938)
EPHB6	Ephrin type-B receptor 6	LDSFLR (aa 756–761) LSSFAF (aa 786–791) LCTFSD (aa 974–979)
BAI2	Adhesion G protein-coupled receptor B2	YPSFLS (aa 1395–1400) FHTFDR (aa 1492–1497) WSTFKS (aa 1544–1549)
BAI3	Adhesion G protein-coupled receptor B3	WDTFKN (aa 1470–1475)
NLGN1	Neuroligin-1	LHTFNT (aa 834–839) FNTFTG (aa 837–842)
NLGN3	Neuroligin-3	YNTFAA (aa 827–832)
NLGN4X	Neuroligin-4	LHTFNT (aa 792–797) FNTFSG (aa 795–800)
LPHN1	Latrophilin-1	ISTFCF (aa 879–884)
UNC5A	Netrin receptor UNC5A	YGTFNF (aa 444–449)
UNC5C	Netrin receptor UNC5C	FGSFNS (aa 533–538)
UNC5D	Netrin receptor UNC5D	FQTFNF (aa 424–429) LDSFGT (aa 670–675)
SLITRK6	SLIT and NTRK-like protein 6	FLSFQD (aa 746–751)
DSC2	Desmocollin-2	WHSFTQ(aa 826–831)
DSC3	Desmocollin-3	WHSFTQ(aa 821–826)
PCDHA1	Protocadherin alpha-1	FITFGK (aa 913–918)
PCDHA2	Protocadherin alpha-2	FITFGK (aa 911–916)
PCDHA5	Protocadherin alpha-5	FITFGK (aa 899–904)
PCDHA6	Protocadherin alpha-6	FITFGK (aa 913–918)
PCDHA7	Protocadherin alpha-7	FITFGK (aa 900–905)
PCDHA8	Protocadherin alpha-8	FITFGK (aa 913–918)
PCDHA9	Protocadherin alpha-9	FITFGK (aa 913–918)
PCDHA10	Protocadherin alpha-10	FITFGK (aa 911–916)
PCDHA11	Protocadherin alpha-11	FITFGK (aa 912–917)
PCDHA12	Protocadherin alpha-12	FITFGK (aa 904–909)
PCDHA13	Protocadherin alpha-13	FITFGK (aa 913–918)
PCDHAC1	Protocadherin alpha-C1	FITFGK (aa 926–931)
PCDHAC2	Protocadherin alpha-C2	FITFGK (aa 970–975)
PCDH9	Protocadherin-9	LSTFAP (aa 1158–1163)
PCDH11X	Protocadherin-11X	LTTFTP (aa 1323–1328)
PCDH11Y	Protocadherin-11Y	LTTFAP (aa 1316–1321)
PCDH8	Protocadherin-8	MSTFCK (aa 984–989)
PCDH10	Protocadherin-10	MPSFVP (aa 955–960) FSTFGK (aa 1000–1005)
PCDH17	Protocadherin-17	FCTFGK (aa 998–1003)
PCDH18	Protocadherin-18	FSTFGK (aa 983–988)
PCDH19	Protocadherin-19	FATFGK (aa 1015–1020)
PCDH12	Protocadherin-12	FQTFGK (aa 1087–1092)
FAT2	Protocadherin Fat 2	LVTFGP (aa 4128–4133)
FAT3	Protocadherin Fat 3	MTTFHP (aa 4263–4268) LSSFQS (aa 4339–4344) FSTFAV (aa 4482–4487)
CELSR3	Flamingo homolog 1	LASFNS (aa 3250–3255)
TMEM132A	Transmembrane protein 132A	FVTFAP (aa 965–970)
TMEM132C	Transmembrane protein 132C	FTTFTT (aa 1056–1061)
TMEM132D	Transmembrane protein 132D	FTTFTA (aa 1047–1052)
TMEM132E	Transmembrane protein 132E	FTTFTT (aa 1015–1020)
ROBO1	Roundabout homolog 1	MKTFNS (aa 1050–1055)
ROBO3	Roundabout homolog 3	LQTFHG (aa 1029–1034)
KIRREL1	Kin of IRRE-like protein 1	YSSFKD (aa 572–577)
NEO1	Neogenin	LKSFAV (aa 1359–1364)

*aa* amino acid.

The indicated residue numbers are based on human protein sequences.

### Axon guidance

**Fig. 2:**
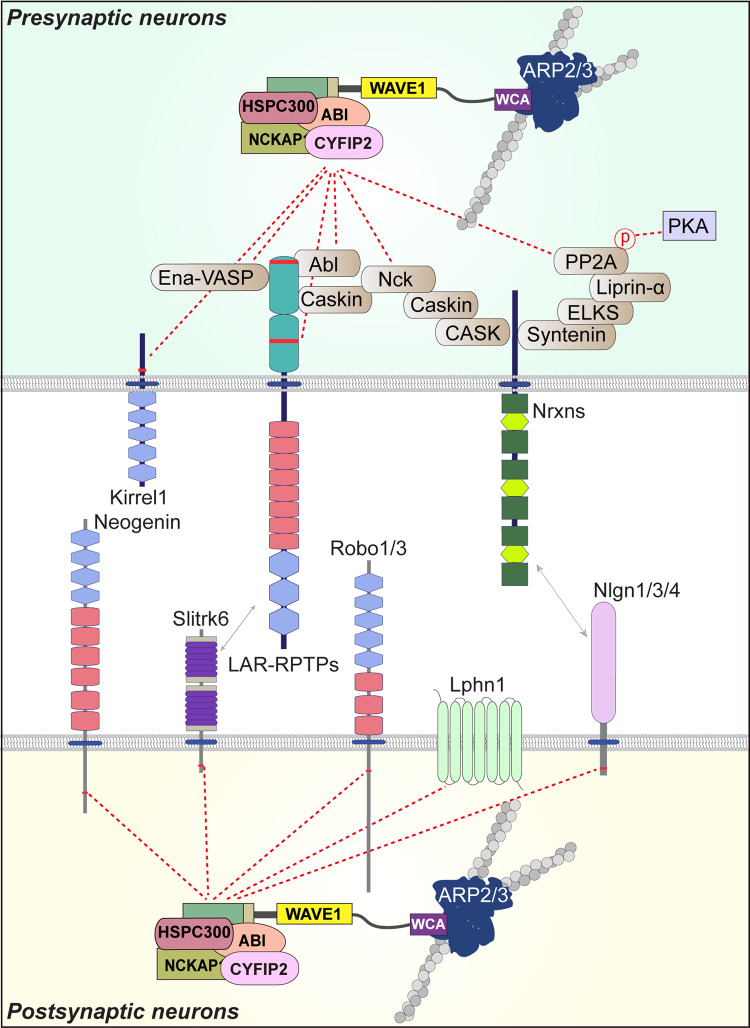
Molecular model of WRC recruitment at neuronal synapses. WRC recruitment is regulated by cell-adhesion proteins and their interacting scaffold proteins at synapses. Various scaffold proteins present in the presynaptic active zone play important roles in WRC recruitment and function. Prominent among them are LAR-RPTPs and Kirrel1. Neurexins (Nrxns) can also bind to the WRC but do not do so directly; instead, they bind indirectly through presynaptic scaffolding proteins, including syntenin, ELKS, liprin-α, PP2A, CASK, Caskins and Nck. In addition to interacting directly with the WRC through their WIRS motif, LAR-RPTPs can also indirectly bind the WRC through interactions with Ena, Abl, and Caskins. Robo receptors, Nlgns, neogenin, Slitrks, and latrophilins are also located at the postsynapse and might recruit the WRC through their WIRS motif.

Accurate formation of synaptic connections during nervous system development requires that axonal growth cones detect a vast array of guidance cues that direct them to their appropriate synaptic targets. These extracellular cues lead to coordinated regulation of actin and microtubule networks. The Arp2/3 complex negatively regulates growth cone translocation and pathfinding but not growth cone morphology^91,92^. In addition, the Arp2/3 complex is required for axon guidance and initiation of growth cone filopodia in *Caenorhabditis elegans* (*C. elegans*) through multiple actin modulatory pathways, including the WRC^93–98^. A number of transmembrane molecules are also linked to the Arp2/3-mediated polymerization of branched actin needed for proper axon guidance in *Drosophila* embryos. Robo1 (roundabout guidance receptor 1) directly interacts with the WRC, which is further modulated by the presence of Slit (a repulsive ligand for the Robo family of receptors in *Drosophila*) secreted from midline glia^99^. The Robo-WRC interaction is essential for Robo1-mediated growth cone repulsion at the midline^99^. In addition, neogenin, a well-known axon guidance regulator, recruits the WRC-Arp2/3 complex to promote actin nucleation and thereby maintain adherens junction dynamics and tension^100^ (Fig. 2). Overall, these studies suggest that by regulating actin cytoskeletal dynamics, the WRC-Arp2/3 axis could act as a central hub for axon guidance. However, whether other axon guidance regulators are also coupled to the WRC-Arp2/3 complex and how they functionally interact in a combinatorial manner in vertebrate neurons remains to be determined.

### Synaptic vesicle clustering and presynaptic assembly

F-actin is highly and preferentially localized around presynaptic vesicle clusters and enriched at the presynaptic active zone, providing a structural substrate for synaptic vesicle clustering, nerve terminal arborization, and axon guidance^101–103^. Intriguingly, synaptic activity promotes presynaptic F-actin assembly, which is essential for proper development of the presynaptic active zone and synaptic vesicle pools^103^. Arp2/3-dependent F-actin networks coordinate the concomitant activation of diverse pathways involving synaptic vesicle proteins, active zone proteins, and/or adapters that dictate synaptic vesicle localization and clustering^102^ (Fig. 2). This WRC component also appears to be involved in synaptic vesicle clustering at nascent synapses through netrin- and Rac1-dependent pathways^51,104^ and synaptic cell-adhesion signaling pathways^105^ in *C. elegans*. Moreover, orthologs of WRC components strongly accumulate in axons, and functional studies using fly lines harboring a null mutation of each component have delineated common abnormalities in axonal and synaptic morphology—specifically, reduced synaptic terminal length at the NMJ—in *Drosophila*^20,70,106–108^. These studies reinforce the idea that the cooperative operation of WRC components in recruiting functional presynaptic machinery involving Arp2/3-mediated actin remodeling is evolutionarily conserved. However, understanding whether and how the WRC contributes to presynaptic assembly, particularly in vertebrate presynaptic neurons, downstream of presynaptic F-actin organization, will require further investigation.

### Transduction of extracellular synaptic adhesion signals to intracellular machinery

Actin dynamics are intimately involved in the regulation of cell surface expression and trafficking of synaptic receptors^109^. The WRC interacts with a host of transmembrane receptors that likely orchestrate *trans*-synaptic adhesion pathways^11,34^. Intriguingly, a highly conserved consensus peptide motif composed of six residues (Φ-x-T/S-F-X-X; Φ = hydrophobic amino acid and X = any amino acid), termed the WIRS (WRC interacting receptor sequence), is present in the cytoplasmic regions of ~115 transmembrane proteins^34^. The WIRS binding surface is occupied by both the Cyfip and Abi subunits of the WRC and is present only in the fully assembled complex^34^. Further analyses have also established that WIRS motifs are prevalent in other potential candidate WRC ligands (Table 1). Because many WIRS-containing proteins are also considered synaptic cell-adhesion molecules (CAMs), these studies suggest the tantalizing concept that various synaptic adhesion pathways, in collaboration with other types of WRC ligands, are involved in the recruitment of the WRC to cellular membranes. Indeed, the WRC forms complexes with the cell-adhesion protein SYG-1 at presynaptic sites and regulates both synaptogenesis and axonal branching in egg-laying motor neurons of *C. elegans*^105^.

Another study reported that *Drosophila* Nlgn1 directly interacts with the WRC through its WIRS motif and regulates NMJ growth and synaptic transmission through its WRC-binding activity^110^. Because vertebrate Nlgns also possess the WIRS motif in their cytoplasmic regions^110^ (Table 1), the regulation of F-actin assembly through the Nlgn-mediated synaptic adhesion pathway is likely an evolutionarily conserved mechanism. Intriguingly, the WRC regulatory pathway appears to integrate cues from other signaling pathways encompassing PKA^62,63,111^. Moreover, a subset of protein kinase pathways, including PKA and postsynaptic cAMP signaling, is essential for compartmentalized signaling at excitatory synapses^112–114^, suggesting the fascinating hypothesis that differentially activated WRC pathways determine the strength of intracellular signals in postsynaptic neurons (Fig. 2). This hypothesis is particularly compelling because synaptic CAMs are thought to mediate the specificity of neural circuit architecture^113,115,116^. Another avenue for future research would be to investigate whether specific paralogs of WRC components align with specific synaptic adhesion pathways. To this end, it would be invaluable to determine the expression patterns of each WRC component in distinct cell types, ideally at single-cell resolution.

## The association of WRC dysfunction with brain disorders

**Fig. 3:**
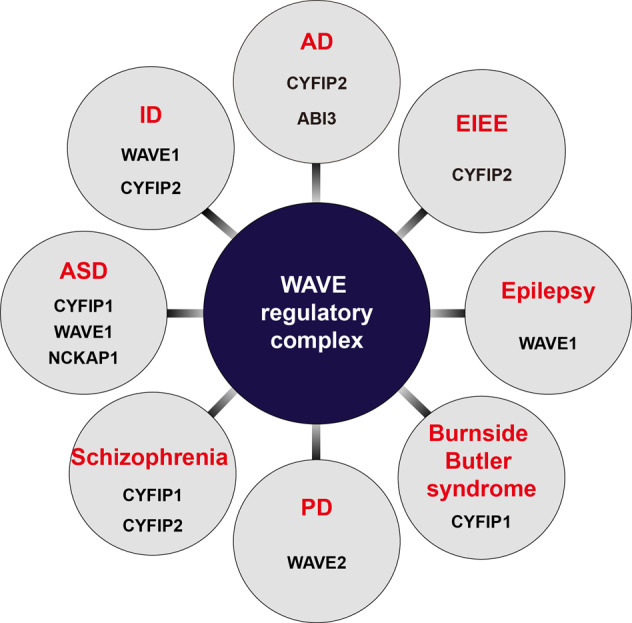
Implication of WRC components in various neurological disorders. Schematic of the neurological disorders discussed in the current paper that are related to dysfunctions of each WRC component. Dysfunction of WAVE1/2, NAP1, ABI3 and CYFIP1/2 might cause neurodevelopmental, neuropsychiatric, and neurodegenerative disorders. ASD autism spectrum disorder, AD Alzheimer’s disease, EIEE early infantile epileptic encephalopathy, ID intellectual disability, PD Parkinson’s disease.

Considering the broad ramifications of the WRC and its interacting networks in governing actin-regulatory machinery in cells, it should come as no surprise that dysfunction in the WRC is associated with a variety of genetic disorders. Because of the universal significance of actin dynamics and remodeling and the fact that mutations affecting the expression and/or other biochemical properties of a single WRC component also influence those of other components, dysregulation of WRC components could manifest as neurological disorders, immune deficiencies, or cancer^11,117^. Here, in keeping with the focus of this review, we primarily discuss links between the dysfunction of WRC components and brain disorders (Fig. 3).

### Disturbance of Cyfip1 functions linked to neurodevelopmental and neuropsychiatric diseases

Deletions in the 15q11.2 region of the human genome (15q11.2 microdeletion), also called Burnside Butler syndrome, are a rare chromosomal anomaly clinically associated with developmental delay, mental retardation, epilepsy, autism spectrum disorder (ASD), schizophrenia, and congenital heart defects^118^. One of the genes within this chromosomal locus is *CYFIP1*^119,120^. Because homozygous deletion of *Cyfip1* in mice is embryonic lethal, most animal studies on this syndrome have primarily been performed using *Cyfip1*^+/−^ mice. These mice exhibit impairments in motor learning and coordination, altered sensory motor gating, aberrant sensory perception/novelty seeking, and enhanced extinction of inhibitory avoidance—phenotypes that are frequently found in individuals with ASD and schizophrenia^26,121–123^. Conversely, transgenic mice overexpressing *Cyfip1* exhibit a series of distinct behavioral phenotypes, including increased fear with mild learning and memory deficits but no ASD-like behavioral abnormalities^124^. These studies suggest that altered *CYFIP1* dosage contributes to divergent endophenotypes associated with diverse neurodevelopmental and neuropsychiatric disorders. Although incompletely understood, abnormalities in NMDAR or mTOR (mammalian target of rapamycin) signaling and altered white matter changes (e.g., thinning of myelin sheath in the corpus callosum) have been proposed as possible pathophysiological mechanisms underlying Cyfip1 dysfunction-associated brain diseases^26,30,122,125^. However, it remains to be determined how Cyfip1 dysfunction-related mechanisms cause alterations in specific neural circuits responsible for specific behavioral domains associated with the indicated neurological disorders.

### Cyfip2 mutations in early infantile epileptic encephalopathy

As described in the previous section (*Role of WRC components during nervous system development*), Cyfip1 and Cyfip2 perform different neuronal functions, as supported by distinct interactome profiles and spatiotemporal expression patterns in the brain^36,126^. Consistent with this, deletions in the chromosomal region (5q33.3–5q35.1) harboring *CYFIP2* have been observed in patients with symptoms distinct from those with *CYFIP1* mutations^117^. In addition, reduced CYFIP2 levels were reported in patients with schizophrenia and AD (Alzheimer’s disease)^127,128^. Intriguingly, de novo Arg87-residue *CYFIP2* variants are associated with various facets of neurodevelopmental disorders, including EIEE (early infantile epileptic encephalopathy)^129–131^. Structural studies have demonstrated that Arg87 *CYFIP2* variants likely disrupt hydrogen bonding between CYFIP2 and WAVE1 or Nap1, leading to structural instability of the WRC and dysregulation of Rac1-mediated WRC activity^132^. Intriguingly, *Cyfip2*^+/R87C^ knock-in mice recapitulate a variety of neurological phenotypes that resemble symptoms of patients with West syndrome^133^. Because West syndrome and Ohtahara syndrome are categorized as subtypes of EIEE^134^—both of which are known to involve presynaptic defects^135^—it will be interesting to examine how the reported functions of CYFIP2 are linked to their pathogenesis mechanisms. Other *CYFIP2* missense variants, notably including presumably pathogenic variants at the Asp724 residue, exhibit variable clinical phenotypes^130^. Moreover, mice with a Cyfip2 deficiency display visual impairments that are frequently observed in individuals with intellectual disability^136^. Determining how these phenotypes are caused by Cyfip2 dysfunction will require future studies that integrate the various cellular and clinical phenotypes observed in *Cyfip2* KO and/or missense variants.

### Dysfunctions of WAVE are linked to neurodevelopmental and neurodegenerative disorders

Exome sequencing and whole-genome sequencing have also identified a number of de novo truncating or missense variants of *WAVE1* in patients with various neurodevelopmental symptoms^137–140^. Certain WAVE1 variants were predicted to disrupt the WCA domain^11^. However, systematic analyses to determine whether the reported *WAVE1* variants are pathogenic in the regulation of actin polymerization processes in the nervous system are currently lacking. WAVE2 is also suggested to be involved in PD (Parkinson’s disease)^141^. WAVE2 interacts with LRRK2 (leucine-rich repeat kinase 2), a key culprit involved in the pathogenesis of PD^141^. LRRK2 phosphorylates WAVE2, stabilizes its levels and modulates the dynamics of WAVE2-mediated phagocytic activity of macrophage cells^141^. Several subsequent reports appear to support the association of WAVE2 with PD^142,143^, although the mechanistic basis of WAVE2 action in the pathogenesis of PD is still unclear.

### Association of Abi3 with Alzheimer’s disease

Rare coding variants of *ABI3* were initially shown to be linked to AD^144^, a relationship that appears to find consistent support in follow-up studies^145,146^. Moreover, deletion of *Abi3* exacerbates various pathophysiological features in a mouse model of AD^147^. ABI3 was also recently proposed as an early biomarker for AD^148^. Overall, a series of these studies have strongly implicated ABI3 in AD pathogenesis, likely through its involvement in microglial motility and/or phagocytosis, in relation to microglial migration into amyloid plaques^149,150^. Whether ABI3 is also involved in other neurodegenerative disorders remains to be determined.

### Implication of NAP1/NCKAP1 in neurodevelopmental disorders

Several heterozygous de novo and ultrarare deleterious variants of *NAP1* have been reported in individuals with various symptoms found in neurodevelopmental disorders^151^. In support of this observation, NAP1 loss of function induces defective neuronal differentiation and abnormal neuronal migration in mice^73,151^. Although these observations provide compelling evidence that Nap1 could be a contributing factor to neurodevelopmental disorders with an ASD core, it is unclear whether *NAP1* variants indeed lead to dysregulation of the WRC. Intriguingly, NAP1 appears to exhibit cell-type–specific expression in the developing human brain^151^. Given the low correlation between mRNA and protein levels in the brain, future work is warranted to determine whether cell-type–specific expression patterns of *Nap1* mRNA are recapitulated at the protein level.

## Outlook and future avenues

The significance of the WRC in regulating various structural and functional aspects of nervous system development and synapse formation has been clearly recognized. However, to fully understand WRC mechanisms, researchers will need to address a number of questions. For example, how is the WRC activated and/or clustered in response to different patterns of extracellular signals in neurons? In particular, do interactions of the WRC with various synaptic adhesion proteins produce distinct *trans*-synaptic signals that converge on a downstream pathway? Does the WRC act similarly in both pre- and postsynaptic neurons? What determines the composition of the WRC in different types of neurons? Does the distinct composition of the presynaptic WRC relate to distinct modes of synaptic transmission by influencing release probability? Do other actin-regulatory pathways (e.g., formins) universally crosstalk with the WRC-mediated Arp2/3 pathway across diverse cell types in various brain areas? Answering these questions will enable researchers to develop various tools for controlling the localization and/or strength of WRC signaling activities, providing an unprecedented opportunity to elucidate the role of the WRC in mediating the specificity and diversity of neural circuit architectures. Given that the Arp2/3 complex is also expressed in nonneuronal cells^152^, it will be illuminating to investigate the nonneuronal roles of each WRC component, ideally using conditional KO mouse lines.
